# Routine angiography in survivors of out of hospital cardiac arrest with return of spontaneous circulation: a single site registry

**DOI:** 10.1186/1471-2261-14-30

**Published:** 2014-03-03

**Authors:** Vishva A Wijesekera, Daniel V Mullany, Catherina A Tjahjadi, Darren L Walters

**Affiliations:** 1 Heart Lung Institute, The Prince Charles Hospital, Brisbane, Australia; 2 Department of Intensive Care, The Prince Charles Hospital, Brisbane, Australia

**Keywords:** Cardiac arrest, Myocardial infarction, OOHCA, Angiography, Therapeutic hypothermia

## Abstract

**Background:**

Coronary revascularization in resuscitated out of hospital cardiac arrest (OOHCA) patients has been associated with improved survival.

**Methods:**

This was a retrospective review of patients with OOHCA between 01/07/2007 and 31/03/2009 surviving to hospital admission. Cardiac risk factors, demographics, treatment times, electrocardiogram (ECG), angiographic findings and in-hospital outcomes were recorded.

**Results:**

Of the 78 patients, 63 underwent coronary angiography. Traditional cardiac risk factors were common in this group. Chest pain occurred in 33.3% pre-arrest, 59.0% were initially treated at a peripheral hospital, 83.3% had documented ventricular tachycardia or ventricular fibrillation, 55.1% had specific ECG changes, 65.4% had acute myocardial infarction (AMI) as the cause of OOHCA and the majority had multi-vessel disease. ST elevation strongly predicted AMI. The in-hospital survival was 67.9% with neurological deficit in 13.2% of survivors. The group of patients who had an angiogram were more likely to have AMI as a cause of cardiac arrest (71.4% vs 40.0%, p = 0.01) and more likely to have survived to discharge (74.6% vs 40.0%, p < 0.01). Poor outcome was associated with older age, cardiogenic shock, longer transfer times, diabetes, renal impairment and a long duration to return of spontaneous circulation.

**Conclusions:**

Acute myocardial infarction was the commonest cause of OOHCA and a high rate of survival to discharge was seen with a strategy of routine angiography and revascularization.

## Background

Out of hospital cardiac arrest (OOHCA) is a leading cause of death in the developed world [1,2]. Coronary artery disease is the cause in up to 90% of cases [3]. Greater than 50% of deaths due to acute myocardial infarction (AMI) occur outside the hospital setting and early ventricular arrhythmias is the most common mechanism of death [4,5], of which ventricular fibrillation is the commonest type. In Australia it is estimated that 15,000 people suffer a cardiac arrest every year, with an incidence estimated at between 9 and 89 per 100,000 person years [6]. The one year survival is estimated at 11.5% [6]. Incorporation of angiography and revascularization into the post resuscitation care of patients with OOHCA and return of spontaneous circulation (ROSC) has been shown in non-randomized case series to be associated with high rates of survival compared to historical controls [7].

The authors’ hospital was one of only two public hospitals that provided acute cardiac catheterization services to an area of approximately 562,000 km^2^ with a population of 1.6 million. In the case of acute ST elevation myocardial infarction (STEMI), due to the large distances involved in regional Queensland, there is no protocol for ambulances to bypass regional hospitals to transport the patient to a percutaneous coronary intervention (PCI) center. The patient is transported to the nearest Emergency Department (ED), where thrombolysis would be considered in the case of STEMI.

The aim of this study was to evaluate the factors associated with survival, in the setting of a strategy that favored routine angiography and revascularization, in patients surviving to hospital admission following OOHCA.

## Methods

**Figure 1:**
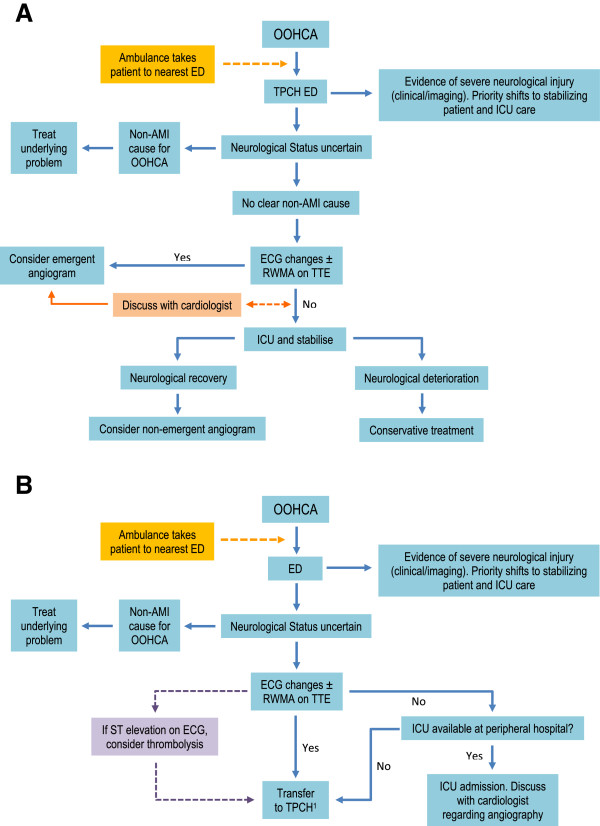
**Protocol used when deciding urgency of coronary angiography. (A)** Flowchart of patients who are transferred direct to TPCH. **(B)** Flowchart of patients who are initially treated at a peripheral hospital. RWMA = regional wall motion abnormalities, TTE = transthoracic echocardiogram (where available). ^1^All patients suspected of an ischaemic cause for OOHCA are urgently transferred to TPCH and the priority of coronary angiography is decided upon arrival (as transfer times may be prolonged depending on distance).

This is a retrospective review of patients with a diagnosis of OOHCA admitted to The Prince Charles Hospital (TPCH). Patients aged 18 years or older admitted to either the coronary care unit or intensive care unit (ICU) during the period 01/07/2007 to 31/03/2009 were included. Patients were identified through departmental databases. Patients who died in the ED and those with non-cardiac causes for OOHCA were excluded. The medical records were reviewed to obtain: demographics, pre-arrest symptoms, ambulance data, initial ECG, blood investigations, examination findings, medications and treatments administered, echocardiographic data, coronary angiographic data, complications in those who had intervention and final diagnosis during hospital stay. Figure 1 summarizes a decision tree followed when evaluating OOHCA patients. Subgroup analyses were performed between those surviving to discharge versus those who did not and those who received coronary angiography versus those who did not. Standard definitions were used where possible (hyperlipidaemia [8], renal impairment [9], cerebral performance category scores [10] and acute myocardial infarction [11]). See accompanying Additional file 1 for detailed methods including details of statistical analyses.

The study is compliant with the Declaration of Helsinki and was approved by the Human Research Ethics Committee at the The Prince Charles Hospital (approval number HREC/13/QPCH/303).

## Results

In the AMI subgroup, 56.9% (29/51 patients) did not have chest pain prior to the event. In those with a non-AMI cause of arrest, 12.5% (3/24 patients) reported chest pain immediately preceding the event where the cause of arrest was myocarditis, dilated cardiomyopathy and cause unknown in one.

**Figure 2:**
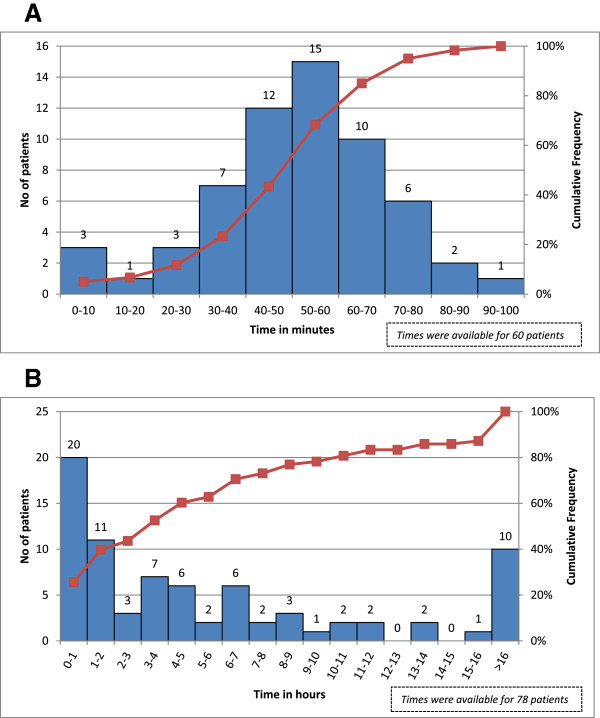
**Transfer times to healthcare facility. (A)** Estimated time between OOHCA and transport to nearest medical facility. **(B)** Estimated time between OOHCA and transport to TPCH.

The mean transfer time to the first hospital (Figure 2A) was 51.7 min (SD ± 20.6 min). Figure 2B shows transport time to TPCH. Those transported direct to TPCH had a median transfer time of 57.6 min (IQR: 49.8-66.0 min), whereas in those initially treated at a peripheral hospital, the median transfer time was 7.2 hours (IQR: 4.2-13.8 hours).

**Figure 3:**
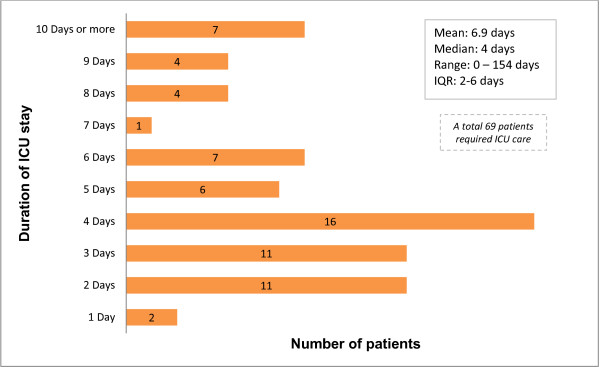
ICU length of stay.

The longest ICU admission of 154 days (Figure 3) was for a patient diagnosed with idiopathic cardiomyopathy with severe ventricular dysfunction as the cause of arrest, and required insertion of a left ventricular assist device and remained in ICU until cardiac transplantation.

### Therapeutic hypothermia in intubated patients

Therapeutic hypothermia (cooling) was administered in 54/65 (83.1%) of intubated patients (status unknown in two patients transferred from elsewhere). In those admitted direct to TPCH 23/30 (76.7%) of intubated patients were cooled; in the seven patients that did not: three regained consciousness immediately after DC cardioversion, one was coagulopathic, one was assessed as already being “cool and shut down”, in one the cause of shock was unclear and in one patient the decision for comfort measures was made early on during resuscitation.

### Access to coronary angiography

In this cohort, 63 patients (80.8%) had coronary angiography performed during the admission. There were 15 patients who did not have angiography. In five, this was because the cause was not thought to be AMI (primary ventricular arrhythmia in one, established dilated cardiomyopathy in four). One patient had a diagnosis of myocarditis based on history, however, died within two hours of arrival in hospital while being prepared for angiography. One patient was known to have severe coronary disease but had refused intervention. The remaining eight patients were assessed to have severe neurological deficit negating any benefit of revascularization (in six of these patients a clinical diagnosis of AMI was made and none of them survived, and in the two other patients the cause of arrest was not identified).

### Direct transfers to TPCH

In direct TPCH admissions, 28/32 (87.5%) had coronary angiography (Table 1). The four who did not were: transferred to another hospital, primary VF/VT, death during preparation for angiogram, and severe neurological deficit. In the 22 patients having emergent angiograms, the median door to needle time was 1.5 hours (IQR: 1.0-2.3 hours). In the six patients who had non-emergent angiograms, the median door to needle time was 64.8 hours or 2.7 days (IQR: 13.9-144.2 hours).

**Table 1:** Patient factors

**Demographics**		**Number**	**Percentage**
**Total out of hospital cardiac arrests**		78	
Males (mean age 61.5 yrs, range 18–84, SD ± 14.9)		63	80.8%
Female (mean age 63.4 yrs, range 34–91, SD ± 16.9)		15	19.2%
Total number of patients with AMI		51	65.4%
STEMI subgroup		31	39.7%
NSTEMI subgroup		20	25.6%
Transfers from peripheral hospitals		46	59.0%
Direct presentations to TPCH		32	41.0%
All coronary angiograms during the same period		6072	
Male		4048	66.6%
- Mean age (in years)		63.0	
Female		2024	33.3%
- Mean age (in years)		64.8	
All STEMI patients^a^		512	
Male		389	76.0%
- Mean age (years)		59.0	
Female		123	24.0%
- Mean age (years)		64.2	
Mean body mass index (BMI) kg/m^2^ (Range 18.8–55.6, SD ± 5.5)		28.5	
Aboriginal or Torres Strait Island origin		1	1.3%
**Risk factors**			
Hypertension		39/77	50.6%
Hyperlipidaemia		28/67	41.8%
Diabetes		13/77	16.9%
Current smoker		22/62	35.5%
Any history of smoking		47/59	79.7%
Known history of ischaemic heart disease		20/76	26.3%
History of previous myocardial infarction		17/76	22.4%
Documentation of illicit drug use at time of cardiac arrest		3/75	4.0%
History of cardiomyopathy		8/77	10.4%
Family history of ischaemic heart disease		23/41	56.1%
Renal failure (GFR <60 ml/min)		34/78	43.6%
**Events immediately following cardiac arrest**			
Chest pain prior to event		25/75	33.3%
Treatment (at the scene): CPR		71/77	92.2%
DCCV		69/78	88.5%
Median time (in min) to ROSC (n = 67), IQR 10.0–27.5		15.0	
Patients with initial treatment at a peripheral hospital		46/78	59.0%
Thrombolysis in ambulance (of the 30 STEMI patients)		2/78	2.2%
Initial rhythm by ambulance	Ventricular fibrillation	59/78	75.6%
	Ventricular tachycardia	6/78	7.7%
	Asystole	4/78	5.1%
	Pulseless electrical activity	4/78	5.1%
	Bradycardia	4/78	5.1%
	Sinus rhythm	1/78	1.3%
	Unknown	0/78	0.0%
Ambulance ECG after ROSC	Unclear	4/60	6.7%
	Atrial fibrillation	6/60	10.0%
	Idioventricular rhythm	2/60	3.3%
	Junctional rhythm	4/60	6.7%
	Sinus rhythm	43/60	71.7%
	Ventricular tachycardia (unavailable and undocumented in 18 patients)	1/60	1.7%
GCS	GCS 3 on arrival of paramedics at the scene	62/77^b^	80.5%
	Brought to hospital intubated or GCS 3	62/78	79.5%
**Characteristics in the emergency department**			
Congestive heart failure		11/78	11.8%
Abnormal left ventricular (LV) function^c^		52/72	72.2%
Cardiogenic shock		34/78	43.6%
Requirement for inotropes		35/78	44.9%
Intubation at any time during the first 24 hours		67/78^d^	85.9%
Insertion of balloon pump		22/78	28.2%
**First ECG in hospital**			
Rhythm:	Sinus rhythm	66/78	84.6%
	Atrial fibrillation	6/78	7.7%
	Ventricular tachycardia	3/78	3.8%
	Ventricular fibrillation	0/78	0.0%
	Pulseless electrical activity	1/78	1.3%
	Junctional rhythm	2/78	2.6%
Specific ECG changes (ST elevation or depression)		43/78	55.1%
Any ST segment changes (including non-specific)		63/78	80.8%
Normal ECG		15/78	19.2%
QRS duration >120 ms		15/78	19.2%
Myocardial territories identified on ECG^e^	Anterior	21/43	48.8%
	Inferior	7/43	16.3%
	Lateral	5/43	11.6%
	Antero-lateral	6/43	14.0%
	Infero-lateral	4/43	9.3%
**Initial treatment in ami patient subgroup**			
Heparin or Enoxaparin		48/51	94.1%
Heparin		42/51	82.4%
Enoxaparin		6/51	11.8%
Aspirin		49/51	96.1%
Clopidogrel		40/51	78.4%
Glycoprotein IIb IIIA receptor blocker		9/51	17.6%
Thrombolysis (by ambulance or hospital)			
As a proportion of all AMI patients		10/51	19.6%
For patients with ST elevation on ECG		10/32	31.3%
**Timing of coronary angiogram**			
Emergent angiogram in entire group		34/78	43.6%
Emergent angiogram in TPCH presentations		22/32	68.8%
Emergent angiogram in peripheral hospital presentations		12/46	26.1%

The denominator is the number of patients with data available.

^a^Refers to all STEMI patients admitted to TPCH during the study period.

^b^One patient was driven to the hospital in a private vehicle.

^c^Initial left ventricular (LV) function estimated by transthoracic echocardiogram, transoesophageal echocardiogram (1 patient) or left ventriculogram during coronary angiogram. Abnormal LV function was recorded if the LV ejection fraction was less than 50% or was recorded as “reduced”. The mean LV function was 37.5%, SD ± 17.9%).

^d^42 of these were intubated at the scene by paramedics.

^e^In those patient who had specific ECG changes.

### AMI patient group

Of the 51 AMI patients, six did not have coronary angiography for the following reasons: five had severe neurological injury and one had known severe coronary disease and declined intervention. All direct transfers to TPCH with STEMI received primary PCI. A total of 34 patients had coronary intervention attempted: 29 patients (85.3%) had bare metal stents placed, four patients (11.8%) had drug eluting stents placed, one patient (2.9%) had a failed procedure, and in one case (2.9%) the type of stent was unknown. One patient who underwent emergency PCI developed pericardial tamponade and died despite being taken for emergency cardiac surgery. This patient had not received thrombolysis but had received dual antiplatelet therapy, heparin and GPIIbIIIa inhibitor therapy. There were no other major bleeding episodes recorded in the other patients, and no other major complications were noted that were attributed to cardiac catheterization.

### Initial ECG in AMI patients who underwent coronary angiography

Table 2 shows ST segment elevation to be highly specific for AMI. Additionally, 19/45 (42.2%) of AMI patients were found to have culprit vessel occlusion (CVO) and this was associated with ST elevation on ECG. No patient with AMI had a normal ECG. In the angiogram group, 25 had a normal or non-specific ECG and eight of these (32.0%) had AMI.

**Table 2:** Initial ECG post arrest and angiographic findings

**Ecg changes in those who had angiogram**	**Number**	**Percentage**
*AMI patients (45 patients)*		
ST elevation (STE)	30/45	66.7%
CVO in patients with STE on ECG	17/30	56.7%
ST depression (STD)	8/45	17.8%
CVO in patients with STD on ECG	1/8	12.5%
Non-specific ECG changes	7/45	15.6%
CVO in patients with non-specific ECG changes	1/7	14.3%
Normal ECG	0/45	0%
CVO in patients with a normal ECG	0/0	0%
*Non AMI patients (18 patients)*		
ST elevation	0/18	0%
ST depression	1/18	5.6%
Non-specific ECG changes	9/18	50.0%
Normal ECG	8/18	44.4%
**Angiogram findings in ami patients (45 patients)**		
Number of vessels diseased^a^		
1 V	13/45	28.9%
2 V	17/45	37.8%
3 V	12/45	26.7%
3 V + LMCA	3/45	6.7%
Culprit vessel (Acute lesions)		
LAD	23/45	51.1%
RCA	5/45	11.1%
LCx	7/45	15.6%
Graft	3/45	6.7%
Unknown	7/45	15.6%
Culprit vessel occlusion	19/45	42.2%
In STEMI subgroup	17/30	56.7%
In NSTEMI subgroup	2/15	13.3%
Recommended treatment based on angiogram		
Follow-on PCI	34/45	75.6%
Recommend CABG	10/45	22.2%
Medical therapy	1/45	2.2%
Patients undergoing immediate PCI to culprit vessel^b^		
Successful PCI^c^	33/34	97.1%

(Total of 63 patients had coronary angiography).

CVO = Culprit Vessel Occlusion, STE = ST segment elevation, STD = ST segment depression.

^a^The denominator here is the total number of AMI patients who had a catheter study (ie. 45 patients).

^b^The denominator here is the number of patients who had follow-on PCI during the index coronary angiogram (34 patients).

^c^Successful PCI was defined as TIMI-III flow and absence of re-occlusion during the index hospital admission.

### Cause of arrest and in-hospital outcomes

Table 3 lists the diagnosed cause of arrest at the end of the hospitalization. Of the 24 patients dying in hospital, the causes were: severe brain injury in 14, cardiogenic shock in seven, shock due to other cause in two and post cardiac surgery in one patient. Of the 34 patients who underwent PCI, 10 (29.4%) died in hospital: in eight the cause of death was neurological injury; in the remaining two the cause was cardiogenic shock.

**Table 3:** Cause of arrest and in-hospital outcomes

	**All patients 78 patients**	**Group A 63 patients**	**Group B 15 patients**
**Cause of OOHCA**			
Acute myocardial infarction	51(65.4%)	45(71.4%)	6(40.0%)
Scar VT/VF	1(1.3%)	1(1.6%)	0(0.0%)
Dilated cardiomyopathy	8(10.3%)	5(7.9%)	3(20.0%)
Primary VF/VT	8(10.3%)	7(11.1%)	1(6.7%)
Myocarditis	3(3.8%)	2(3.2%)	1(6.7%)
Unknown	7(9.0%)	3(4.8%)	4(26.7%)
**In-hospital outcomes**			
Survived to discharge	54/78(69.2%)	48(76.2%)	6(40.0%)
Neurological deficit	7/78(9.0%)		
Neurological deficit (in survivors)	7/54(13.0%)		
AICD recommended	22/78(28.2%)		
AICD recommended (in survivors)	22/53(41.5%)		
Survival in:			
AMI patients	34/51(66.7%)		
AMI patients who had angiogram	33/45(73.3%)		
AMI patients who did not have angiogram	1/6(16.7%)		

Note:

Group A = patients who had coronary angiogram.

Group B = patients who did not have coronary angiography.

### Subgroup analysis

Comparing those who survived to discharge (good outcome) versus those who died during admission (poor outcome), older age (on average, those with a good outcome were eight years younger), diabetes, renal impairment, lower GCS by paramedics, longer transfer time to first hospital, longer transfer time to TPCH, longer duration to ROSC, presence of congestive heart failure and requirement for inotropes were all associated with a poor outcome. Initial treatment at a peripheral center was associated with good outcome, presumably because those with a poor prognosis were not transferred. A shockable rhythm on ECG was associated with a good prognosis. Coronary angiography and aspirin use was associated with a good prognosis which is at least partially attributable to the selection process.

The group who had coronary angiography was compared to the group that did not (Table 4). Those with a longer transfer time to hospital and lower GCS by paramedics were less likely to have coronary angiography (probably reflecting neurological status), and those with a suggestion of AMI (based on ventricular arrhythmias or ECG changes) were more likely to have coronary angiography. The improved survival seen in the group having angiogram may be partly due to the selection process.

**Table 4:** Subgroup analysis

	**Cathed (Group A) 63 patients**	**Non-cathed (Group B) 15 patients**	**p-value**	**Good outcome (Group C) 53 patients**	**Poor outcome (Group D) 25 patients**	**p-value**
**Risk factors**						
Age, mean(±SD)	61.1(14.1)	64.9(19.5)	0.20	59.4(14.9)	67.1(14.7)	0.01
Males, no. (%)	52(82.5)	11(73.3)	0.21	45(84.9)	18(72.0)	0.09
Diabetes, no. (%) [1]*	11(17.5)	2(14.3)	0.39	3(5.7)	10(41.7)	<0.01
Hypertension, no. (%) [1]	32(50.8)	7(50.0)	0.48	27(50.9)	12(50.0)	0.46
Hyperlipidaemia, no. (%) [11]	25(46.3)	3(23.1)	0.07	19(41.3)	9(42.9)	0.45
History of IHD, no. (%) [2]	17(27.4)	3(21.4)	0.33	15(28.8)	5(20.8)	0.23
Serum creatinine μmol/L, median(IQR)	102(86–115)	125(113–161)	<0.01	100(84–114)	119(107–142)	<0.01
GFR ml/min, median(IQR)	63(52–78)	46(37–54)	<0.01	66(55–81)	50(42–60)	<0.01
History of smoking, no. (%) [19]	41(80.4)	6(75.0)	0.37	34(79.1)	13(81.3)	0.42
**Peri cardiac arrest factors**						
Time, min to first hospital, median(IQR) [16]	52(40–61)	70(51–80)	<0.01	50(40–60)	63(50–73)	0.02
Time, hrs to TPCH, median(IQR)	3.9(1.0 -9.3)	4.0(1.9–6.9)	0.14	5.75(1.05–11.8)	1.25(1–4)	<0.01
History of chest pain, no. (%) [3]	21(33.9)	4(30.8)	0.42	16(31.4)	9(37.5)	0.30
Time to ROSC in min, median(IQR) [11]	15(10–25)	22.5(15–29)	0.36	13.5(10–22.2)	20(15–35)	0.01
Treated at a peripheral hospital, no. (%)	35(55.6)	11(73.3)	0.12	35(66.0)	11(44.0)	0.03
**ECG factors**						
Initial rhythm VF/VT, no. (%)	56(88.9)	9(60.0)	<0.01	47(88.7)	18(72.0)	0.03
ST elevation, no. (%)	30(47.6)	2(13.3)	<0.01	21(39.6)	11(44.0)	0.36
QTc in milliseconds, mean(±SD) [2]	453(46)	464(51)	0.23	454(48.4)	457(43.4)	0.42
**Assessments and tests**						
GCS by paramedics, mean(±SD) [2]	5.4(4.7)	3.0(0)	0.03	5.84(5.06)	3.12(0.6)	<0.01
CCF in ED, no. (%)	9(14.3)	2(13.3)	0.46	5(9.4)	6(24.0)	0.04
First measured LVEF, mean(±SD) [6]	37.7(17.2)	36.7(21.6)	0.43	39.2(18.2)	33.5(16.8)	0.11
Initial serum K mmol/L, mean(±SD)	3.9(0.7)	4.2(0.6)	0.06	3.9(0.7)	4.0(0.7)	0.20
Initial serum Mg mmol/l, mean(±SD) [5]	1.0(0.3)	1.0(0.5)	0.49	1.0(0.3)	1.1(0.6)	0.06
Peak CK in IU/L, median(IQR) [9]	1440(524–3562)	1810(1070–4050)	0.15	1440(544–3442)	1810(613–3870)	0.37
Peak Troponin I in μmol/L, median(IQR) [5]	20(4–71)	9(1–35)	0.20	15(5–56)	32(1–75)	0.42
**Treatments**						
Use of inotropes, no. (%)	27(42.9)	8(52.3)	0.24	20(37.7)	15(60.0)	0.03
Heparin/Enoxaparin, no. (%) [1]	59(93.7)	14(100)	0.34	51(96.2)	22(91.7)	0.21
Aspirin, no. (%) [1]	62(98.4)	11(78.6)	<0.01	52(98.1)	21(87.5)	0.03
Clopidogrel, no. (%) [1]	50(79.4)	3(21.4)	<0.01	37(69.8)	16(66.7)	0.39
Balloon pump, no. (%)	20(31.7)	2(13.3)	0.08	15(28.3)	7(28.0)	0.49
ICU stay in days, median(IQR)	4(2–6)	4(2–5.5)	0.36	4(2–6)	4(2–4)	0.15
Coronary angiogram at any time, no. (%)	N/A	N/A	N/A	47(88.7)	16(64.0)	<0.01
**Initially presenting to TPCH**				18 patients	14 patients	
Emergent coronary angiogram, no. (%)				11(61.1)	11(78.6)	0.15
Coronary angiogram at any time, no. (%)				16(88.9)	12(85.7)	0.40
**Outcomes**						
OOHCA caused by AMI, no. (%)	45(71.4)	6(40.0)	0.01			
Survived to discharge, no. (%)	47(74.6)	6(40.0)	<0.01			
Neurological deficit, no. (%)	7(11.1)	0(0.0)	0.09			
Neurological deficit in survivors, no. (%)	7/47(14.9)	0/6(0.0)	0.16			

*[n] Number of patients for whom information was missing is represented within square brackets.

Group A = patients who had coronary angiogram (“cathed patients”).

Group B = patients who did not have coronary angiography (“non-cathed patients”).

Group C = patients who survived to discharge from hospital.

Group D = patients who died during the hospital admission.

### Multivariate logistic regression

Variables known to be associated with poor outcomes together with those variables from the univariate analysis with p <0.1 were included. The final model was limited to a maximum of five variables due to the limited number of events (32). Age was centered on its median value in the sample (64 years). Glasgow coma score (GCS) in the ED was the most important predictor of a good outcome in that no patient who arrived in the ED with a GCS of >8 died. Exact logistic regression was used to assess the association of GCS with good outcome. The median unbiased estimate of the effect of ED GCS >8 compared to the intubated patient on good outcome was an odds ratio of 13.6 with a very large 95% confidence interval (2-∞). There was no difference in outcome between the intubated group and those that had a GCS ≤8 in the ED.

When ED GCS was included in a standard logistic regression model, the fact that there were no events in the GCS >8 group rendered the model uninformative. Variables other than GCS were included in a standard logistic regression model. In summary, lower age, the absence of cardiogenic shock, a transfer from another hospital and not being cooled were associated with the outcome (survival without neurological injury). Not being cooled was confounded on the better ED GCS. Once again, it is likely that the transferred patients represented a better prognostic group.

Standard multivariate logistic regression diagnostics was performed. The Hosmer Lemeshow goodness of fit test was not significant (p = 0.46) and the area under the receiver operating characteristic curve was 0.81 even when the ED GCS was not included Please see accompanying Additional file 2 for tabular data.

## Discussion

Ischaemic heart disease is the most common substrate in OOHCA and therefore the demographics reflect a high incidence of cardiac risk factors as has been reported elsewhere [6,12,13]. This study cohort represents those with a better prognosis partly due to selection of those who survived to hospital admission. Our immediate success rates for PCI was 97.1% compared to 76% reported by Spaulding [14]. When we identified a culprit lesion, this was located proximal or mid vessel, which has been shown to be a strong predictor of cardiac arrest [15] – possibly related to a larger area of ischaemic myocardium. Anyfantakis, in a study of routine emergency coronary angiography in unselected survivors of OOHCA, found that 37.5% had angiographic or clinical evidence of AMI as a cause for the cardiac arrest [16]. They found ST elevation on the admission ECG to be a strong independent correlate of AMI and suggested this as a method of triaging patients for emergent cardiac catheterization, which our findings support.

Despite a high incidence of AMI in this study, chest pain was documented in only 33%, although, anoxic brain injury may cause amnesia to pre-arrest symptoms. Spaulding et al found that ST elevation and chest pain to be poorly predictive of acute coronary occlusion [14] in the OOHCA setting, hence, the absence of these should not preclude coronary angiography. Our finding of 12.5% of patients with ST depression and 14.3% of patients with non-specific ECG changes having culprit vessel occlusion (Table 2) supports this. ST segment elevation on initial ECG is highly predictive of an ischaemic etiology, and the International Liaison Committee on Resuscitation recommends such patients undergo immediate coronary angiography [17].

The Parisian Regional Out of hospital Cardiac Arrest Trial, enrolled 435 patients who had no obvious extracardiac cause for OOHCA and all underwent immediate coronary angiography and PCI if indicated [18]. At least one significant coronary artery lesion was found in almost all patients with STEMI on ECG (128/134; 96%) and in a significant proportion of patients without STEMI (176/301; 58%). PCI was successful in 99/128 STEMI patients, and in 78/176 patients without STEMI. Hospital survival was 40%. Multivariate analysis confirmed that a successful PCI was an independent predictor of survival irrespective of the initial post-resuscitation ECG (odds ratio 2.06; 95% CI 1.16–3.66). Spaulding [14] also found successful PCI to be an independent predictor of survival. Bendz [19] showed that PCI in a select group of patients with STEMI following resuscitated OOHCA had a good outcome with both an in-hospital and two year survival of 72.5%. Others have shown a survival benefit in those undergoing coronary angiography versus those who did not - 67% vs 18% in some studies [20].

A meta-analysis by Larsen et al found a high prevalence of significant coronary disease in patients after resuscitated OOHCA, and they recommend consideration of emergent coronary angiography in the absence of a non-cardiac cause for arrest [21]. Zanuttini [7] found that in a group of resuscitated OOHCA patients, there was a high incidence of coronary disease and ST elevation on ECG predicted culprit coronary lesions. They also found that emergency angiography and successful PCI was independently related to in-hospital mortality and survival after OOHCA. Similar to our findings, these investigators also found that the absence of ST elevation should not prevent a patient having coronary angiography. Sunde [22] has shown that incorporation of a standardized post-cardiac arrest protocol which included PCI (where clinically indicated) was associated with improved hospital discharge rates, neurological outcome and one year survival compared to historical controls.

The 2010 International Consensus on Cardiopulmonary Resuscitation and Emergency Cardiovascular Care Science with Treatment Recommendations [23] as well as the European Resuscitation Council guidelines [24], recommend that coronary angiography be considered in the post cardiac arrest patient. In our experience this strategy of routine angiography in selected patients was associated with a high rate of survival to discharge (67.9%) and survival without significant neurological impairment (86.8% of survivors). In the direct admissions to TPCH, even those having non-emergent angiography, the median door to needle time of 2.7 days is in keeping with our policy of performing the angiogram early on in the admission.

We found coma to be a common feature in patients following resuscitated OOHCA and this should not delay revascularization. Neurological prognostication is difficult early in the course of OOHCA. The decision not to perform emergent coronary angiography was based on clinical evaluation that included factors such as greater than 60 min to ROSC, advanced age, poor pre-morbid functional status, history of severe cognitive impairment, asystole as the presenting rhythm and presence of an obvious non-ischaemic cause for arrest. With our strategy of routine angiography in these patients, we did not find therapeutic hypothermia to be an obstacle, and the overall procedural complication rate was low.

### Limitations

The small sample size and retrospective nature of the study causes selection bias. The pre-hospital setting was poorly documented (especially in those transferred from elsewhere). It was difficult to accurately ascertain how many OOHCA were witnessed and, in some cases, time to ROSC. The incorporation of patients transferred from other centers introduced selection bias as only those with good prognosis are expected to have been transferred. It follows therefore, that those transferred from peripheral centers should not be denied coronary angiography (if clinically indicated). Using the published incidence of OOHCA in Australia [6], the patients treated at this institution (as a percentage of the estimated total in the population serviced by TPCH), ranged from as low as 11% to as high as 100%. The lower estimate would suggest that a significant number are not being transferred, presumably related to poor prognosis or death, however, such wide-ranging estimates are difficult to interpret.

## Conclusions

Most survivors of OOHCA have multi vessel coronary disease and AMI is the leading cause of arrest. Absence of symptoms or ST elevation on ECG should not delay or prevent coronary angiography. In our experience, in patients surviving to hospital admission following OOHCA, a strategy of routine coronary angiography and revascularization is safe and associated with a high rate of survival to discharge.

## Abbreviations

OOHCA: Out of hospital cardiac arrest; ECG: Electrocardiogram; AMI: Ccute myocardial infarction; TPCH: The Prince Charles Hospital; STEMI: ST segment elevation myocardial infarction; ROSC: Return of spontaneous circulation; ICU: Intensive care unit; VT: Ventricular tachycardia; VF: Ventricular fibrillation; TTE: Transthoracic echocardiogram; PCI: Percutaneous coronary intervention; CVO: Culprit vessel occlusion; GCS: Glasgow coma scale; OR: Odds ratio; CI: Confidence interval; IQR: Interquartile range; SD: Standard deviation; GFR: Glomerular filtration rate (in ml/min); ∞: Infinity; BMI: Body mass index; CPR: Cardiopulmonary resuscitation; LV: Left ventricle; LAD: Left anterior descending coronary artery; RCA: Right coronary artery; LCx: Left circumflex coronary artery; LMCA: Left main coronary artery; CABG: Coronary artery bypass graft; K: Potassium; Mg: Magnesium; CK: Creatine kinase.

## Competing interests

The authors have no competing interests.

## Authors’ contributions

VW was involved in data collection, analysis and drafting of the manuscript. DM participated in the design of the study and performed the statistical analysis as well as helped drafting of the manuscript. CT was involved in data collection and helped with drafting of the manuscript. DW conceived the study, participated in the design and helped draft the manuscript. Both CT and DW were involved in critiquing the article.

## Authors’ information

Vishva A. Wijesekera, FRACP

Daniel V. Mullany, MMedSc, FANZCA, FCICM

Catherina A. Tjahjadi, MBBS

Darren L. Walters FRACP, FCSANZ, FSCAI, MPhil, Grad Cert Mang

DW is the director of the cardiac catheter laboratory, the executive chair of the Heart Lung Institute and the executive director of the Prince Charles Hospital. DM is an intensive care specialist at The Prince Charles Hospital. VW and CT are cardiology trainees at this institution.

## Pre-publication history

The pre-publication history for this paper can be accessed here:

http://www.biomedcentral.com/1471-2261/14/30/prepub

## Supplementary Material

Additional file 1 Further details of study methods. Click here for file

Additional file 2 Multivariate analysis. Statistical analysis tables. Click here for file
