# Supplementary material for: Routine angiography in survivors of out of hospital cardiac arrest with return of spontaneous circulation: a single site registry
Source: BMC Cardiovasc Disord. 2014 Mar 3;14:30. doi: 10.1186/1471-2261-14-30 (PMC3944915; doi:10.1186/1471-2261-14-30)
Supplement: Additional file 1 — Further details of study methods. [file 1471-2261-14-30-S1.pdf]

SUPPLEMENT 1

Detailed methods

The following are the definitions of the variables studied:

1. Demographics: date of birth, gender, indigenous status (Australian aboriginal vs not), height, weight, date of OOHCA (out of hospital cardiac arrest)
2. Pre-arrest symptoms (usually from ambulance records): chest discomfort or dyspnea
3. Ambulance data: Glasgow coma scale (GCS) on arrival of the paramedics, initial ECG rhythm: ventricular tachycardia (VT), ventricular fibrillation (VF) or other, bystander CPR, requirement for electrical DC current cardioversion (DCCV) by paramedics, estimated time to return of spontaneous circulation (ROSC) in minutes (defined as the presence of a detectable carotid or femoral pulse and a measurable blood pressure by the attending paramedic staff), initial transfer hospital, thrombolysis by paramedics. Detailed ambulance ECG data including rhythm, ST segment changes (where available)
4. First ECG done at the receiving emergency department (ED): rhythm, ST changes, presence of left bundle branch block (LBBB), corrected QT interval in milliseconds (QTc).
5. Initial serum potassium and magnesium level (mmol/L). Peak Troponin I (in µmol/L) and creatinine kinase (CK) levels (in IU/L) in the peri-arrest period were recorded.
6. GCS or intubation status at the time the patient was brought to the ED.
7. Initial examination in hospital: presence of pulmonary oedema, initial left ventricular function assessment (LVEF), presence/absence of shock, requirement for inotropes.
8. Initial treatment in the emergency department: Heparin/Enoxaparin, Glycoprotein IIbIIIa inhibitor use, administration of Aspirin and/or Clopidogrel, thrombolysis in the emergency department
9. Intubation and ventilation at any time in the first 24 hours after the cardiac arrest (includes patients who were brought to hospital not intubated, and then intubated in ED)
10. Requirement for an intra-aortic balloon pump on initial assessment
11. Intensive care unit (ICU) length of stay in days
12. First formal assessment of LV function (echocardiogram or left ventricular angiogram)
13. Coronary angiography: Whether the patient had an angiogram, and if not the reasons for not doing so. If performed, whether it was an emergent or non-emergent angiogram. Door to catheter laboratory table and door to needle time were recorded.
14. In those who did not have coronary angiography performed – whether any other assessment of coronary vasculature/perfusion was made: Computer tomographic coronary angiography (CTCA) or cardiac magnetic resonance imaging (C-MRI)
15. Angiogram findings recorded were: (1) Culprit vessel (as indicated by the operator) and the degree of stenosis. (2) The site of culprit lesion was categorized as proximal, mid or distal vessel. (3) Culprit vessel occlusion or not. (4) Number of vessels with coronary disease. (5) Decision for follow-on percutaneous coronary intervention (PCI) and success of PCI (as recorded by the interventionalist or recording of TIMI 3 flow in the culprit vessel post PCI). (6) Type of stent (bare metal versus drug eluting). If there was no obvious culprit vessel identified this was recorded as such.
16. Culprit vessel occlusion was defined as the presence of an acute 100% lesion in the coronary artery thought to be responsible for the myocardial infarct.
17. Complications of the procedure – including major bleeding requiring blood transfusion, vascular complications, death related to the procedure, or any other recorded complication during that admission.
18. Final diagnosis of cause of OOHCA at the end of the hospital admission based on discharge summary, clinical notes and/or death certificate. Patients with myocardial infarction were further sub-categorised into ST segment elevation myocardial infarction (STEMI) and non-ST segment elevation myocardial infarction (NSTEMI).
19. Survival and neurological deficit (see below for definition).
20. Recommendation of automatic implantable cardioverter defibrillator (AICD) as per current guidelines and if recommended whether the patient had this procedure during the index admission or not.

The timing of coronary angiography was decided by the physicians assessing the patients in the ED in consultation with the cardiology service where appropriate. If the patient had been transferred from a peripheral hospital to the TPCH ICU, an assessment was made in ICU.

An emergent coronary angiogram, refers to the decision to perform the coronary angiogram as an emergency procedure. This would have had to be made at the point of initial assessment at TPCH and documentation in the medical notes to reflect this including emergency activation of the catheter laboratory. If on the other hand, it was unclear from the medical notes that the catheter laboratory was activated "for an emergency procedure", we recorded this as a non-emergent coronary angiogram – even if the coronary angiogram was performed within 24 hours of the patient presenting to TPCH.

ECG changes, were grouped into specific changes (ST segment or T-wave changes in a coronary territory that was suggestive of an acute coronary artery lesion), non-specific changes (when the aforementioned ECG changes did not identify a particular vascular territory and also any subtle changes such as isolated changes in a single lead) and a normal ECG.

ICU care involved standard protocols and included therapeutic hypothermia (cooling) unless a contraindication existed.

Data Collection
In the tables provided, the numbers enclosed within square brackets denote the total number of patients who had information available for that particular variable.

The various cardiac risk factors were recorded as follows:
Hyperlipidaemia – Presence or absence was recorded as in medical records, however if this was not mentioned and no fasting cholesterol measurement was available on the hospital pathology database (AusLab®) the field was left blank. A fasting total cholesterol of >6.2 mmol/L was considered abnormal and recorded as the presence of hyperlipidaemia[1].

Diabetes – A previously established diagnosis, a new diagnosis during the index admission, or if not specifically mentioned, if the patient was treated with oral hypoglycaemics or insulin either prior to or at any time during the admission.

Smoking history was recorded based on what was recorded in the medical records. This was subdivided into the current smokers, reformed smokers (if the patient had any history of smoking) and non-smokers.

Hypertension – The diagnosis of hypertension was based solely on a previous diagnosis as mentioned in the medical records, or the recording of a new diagnosis of hypertension by the clinicians. As standard treatment of myocardial infarction involved beta-blockers and ACE inhibitors, the presence of these medications on the patient's medication list in hospital did not automatically lead to a recording of "hypertension" in their risk factor profile.

Family history of premature coronary disease (CAD): CAD in a male first degree relative under 55 years or in a female first degree relative under 65 years, as defined in the Framingham study[2], however, as these details were not always specifically mentioned in the clinical notes, any documentation of "positive family history" was included.

Renal function (glomerular filtration rate, GFR): This was calculated using the highest eGFR (electronically calculated glomerular filtration rate, measured in ml/min, as displayed on the hospital laboratory database AusLab) in the first 72 hours after the cardiac arrest. Any patient with an eGFR <60 ml/min (stage 2 chronic kidney disease) was recorded as having renal impairment[3].

Illicit drug use – If not explicitly recorded in the notes, it was assumed that there was no significant history of drug use contributing to the cardiac arrest.

Presence or absence of a personal history of ischaemic heart disease or cardiomyopathy was recorded solely based on what was available in the patient's medical records.

Neurological deficit was defined as the documentation of any cognitive deficit or gross motor weakness at the time of discharge that was new and attributed to the cardiac arrest. This was then categorized using the Cerebral Performance Category scores[4] and recorded as satisfactory neurological status if a score of 1 or 2 was obtain and any higher scores were recorded as the presence of neurological deficit.

Definition of AMI

The definition of AMI met the criteria specified in the third universal definition of myocardial infarction[5] published in 2012:

1. Those with 100% occluded coronary vessels (thought to be "acute occlusion" by the operator)
2. Those with ECG changes (ST segment elevation or depression) in a specific territory with a Troponin rise (and if the patient had a catheter study with a vessel lesion identified as "culprit" on the catheter report)
3. Those with regional wall motion abnormalities seen on echocardiogram and a rise in Troponin (in those who did not have angiography), and the treating clinician concluded that the cause of arrest was AMI.
4. Any other patient who had an angiogram who did not have an occluded vessel, but the operator concluded myocardial infarction as the diagnosis and defined a culprit vessel based upon TIMI flow within that vessel and/or ECG changes.

Two flowcharts are provided (Figure 1A and Figure 1B) which illustrate decision tree followed in this region when deciding where the patient is taken for treatment and the urgency of coronary angiography. We stress that this is a guide only, and at any stage, the treating clinician can overrule the process outlined in the flowchart.

Statistical Analysis

Normally distributed continuous variables were expressed as mean and standard deviation (SD) otherwise as median and the inter-quartile range (IQR). Continuous variables were compared using Students 't' test or Mann-Whitney U test. Categorical variables were compared using chi-square test or Fisher's exact test. A two sided p value of ≤ 0.05 was considered significant. No adjustments were made for multiple comparisons. Exact logistic regression was used when categorical independent variables contained few events. The calculations were performed using STATA software (Statacorp College Station, Texas) Version 11.

REFERENCES FOR SUPPLEMENT 1:

1. National Cholesterol Education Program: **Third Report of the National Cholesterol Education Program (NCEP) Expert Panel on Detection, Evaluation, and Treatment of High Blood Cholesterol in Adults (Adult Treatment Panel III) final report.** *Circulation* 2002, **106:**3143-3421.
2. Parmar MS: **Family history of coronary artery disease--need to focus on proper definition!** *European heart journal* 2003, **24:**2073.
3. National Kidney Foundation: **K/DOQI clinical practice guidelines for chronic kidney disease: evaluation, classification, and stratification.** *American journal of kidney diseases : the official journal of the National Kidney Foundation* 2002, **39:**S1-266.
4. Jacobs I, Nadkarni V, Bahr J, Berg RA, Billi JE, Bossaert L, Cassan P, Coovadia A, D'Este K, Finn J, Halperin H, Handley A, Herlitz J, Hickey R, Idris A, Kloeck W, Larkin GL, Mancini ME, Mason P, Mears G, Monsieurs K, Montgomery W, Morley P, Nichol G, Nolan J, Okada K, Perlman J, Shuster M, Steen PA, Sterz F, et al: **Cardiac arrest and cardiopulmonary resuscitation outcome reports: update and simplification of the Utstein templates for resuscitation registries: a statement for healthcare professionals from a task force of the International Liaison Committee on Resuscitation (American Heart Association, European Resuscitation Council, Australian Resuscitation Council, New Zealand Resuscitation Council, Heart and Stroke Foundation of Canada, InterAmerican Heart Foundation, Resuscitation Councils of Southern Africa).** *Circulation* 2004, **110:**3385-3397.

5. Thygesen K, Alpert JS, Jaffe AS, Simoons ML, Chaitman BR, White HD, Katus HA, Lindahl B, Morrow DA, Clemmensen PM, Johanson P, Hod H, Underwood R, Bax JJ, Bonow RO, Pinto F, Gibbons RJ, Fox KA, Atar D, Newby LK, Galvani M, Hamm CW, Uretsky BF, Steg PG, Wijns W, Bassand JP, Menasche P, Ravkilde J, Ohman EM, Antman EM, et al: **Third universal definition of myocardial infarction.** *Circulation* 2012, **126:**2020-2035.
