# Supplementary material for: Routine angiography in survivors of out of hospital cardiac arrest with return of spontaneous circulation: a single site registry
Source: BMC Cardiovasc Disord. 2014 Mar 3;14:30. doi: 10.1186/1471-2261-14-30 (PMC3944915; doi:10.1186/1471-2261-14-30)
Supplement: Additional file 2 — Multivariate analysis. Statistical analysis tables. [file 1471-2261-14-30-S2.pdf]

SUPPLEMENT 2

Multivariate analysis

Good Outcome	OR	SE	z	P>z	95% CI
Age	0.95	0.02	-2.84	0.004	0.91 - 0.98
Cardiogenic shock	0.34	0.19	-1.97	0.049	0.12 - 0.99
Transfer from another hospital	3.26	1.79	2.15	0.032	1.11 - 9.57
Cooling	0.26	0.16	-2.12	0.034	0.07 - 0.90
Constant	2.92	1.94	1.61	0.107	0.79 - 10.72

.

Good Outcome	OR	Suff	2*Pr(Suff.)	95% CI
Intubated	1			
GCS <=8	0.80	11	0.86	0.26 - 2.48
** median unbiased estimates (MUE)*				

OR = odds ratio
SE = standard error
CI = confidence interval
